# *Alpinia pricei* Rhizome Extracts Induce Cell Cycle Arrest in Human Squamous Carcinoma KB Cells and Suppress Tumor Growth in Nude Mice

**DOI:** 10.1093/ecam/nep142

**Published:** 2011-06-18

**Authors:** You-Cheng Hseu, Chih-Sheng Chen, Sheng-Yang Wang

**Affiliations:** ^1^Department of Cosmeceutics, College of Pharmacy, China Medical University, 91 Huseh-Shih Road, Taichung 40402, Taiwan; ^2^Institute of Nutrition, China Medical University, Taichung 40402, Taiwan; ^3^Department of Forestry, National Chung Hsing University, Taichung 40402, Taiwan

## Abstract

*Alpinia pricei* has been shown to induce apoptosis in human squamous carcinoma (KB) cells. In this study, we report the effectiveness of the ethanol (70%) extracts of *A. pricei* rhizome (AP extracts) in terms of tumor regression as determined using both *in vitro* cell culture and *in vivo* athymic nude mice models of KB cells. We found that the AP extract (25–200 **μ**g/mL) treatment decreased the proliferation of KB cells by arresting progression through the G2/M phase of the cell cycle. This cell cycle blockade was associated with reductions in cyclin A and B1, Cdc2, and Cdc25C, and increased p21/WAF1, Wee1, p53 and phospho-p53 (p-p53) in a dose- and time-dependent manner. Moreover, we found that AP extract treatment decreased metalloproteinase-9 (MMP-9) and urokinase plasminogen activator (u-PA) expression, while expression of their endogenous inhibitors, tissue inhibitor of MMP-1 (TIMP-1) and plasminogen activator inhibitor-1 (PAI-1), were increased in KB cells. Furthermore, AP extract treatment effectively delayed tumor incidence in nude mice inoculated with KB cells and reduced the tumor burden. AP extract treatment also induced apoptotic DNA fragmentation, as detected by *in situ* TUNEL staining. Thus, *A. pricei* may possess antitumor activity in human squamous carcinoma (KB) cells.

## 1. Introduction

*Alpinia pricei* Hayata (family Zingiberaceae) is a perennial rhizomatous herb indigenous to Taiwan. Rhizomes of the Zingiberaceae family are vegetables widely used in many Asian countries, and their medicinal functions are broadly discussed and accepted in many traditional recipes. *Alpinia* plants possess antioxidant [1], anti-inflammatory [2], anticancer [3, 4], immunostimulatory [5], hepatoprotective [6] and antinociceptive [7] activities. Furthermore, the rhizomes of *Alpinia* species (*galanga*) are used to treat problems associated with the digestive system and to relieve symptoms of bronchitis, measles, rubella and cholera [8]. Traditional medicine in Algeria has used the roots of *Alpinia* species for the treatment of respiratory infections for centuries [5]. *Alpinia pricei* is also used in traditional Chinese medicine to treat abdominal distension and increase stomach secretion and peristalsis [9, 10]; however, very few biological activity tests are reported.

Chemoprevention, which refers to the administration of naturally occurring agents to prevent initiation and promotion events associated with carcinogenesis, is being increasingly appreciated as an effective approach for the management of neoplasia [11]. Many studies show associations between abnormal cell cycle regulation and apoptosis and cancer, in as much as the cell cycle inhibitors and apoptosis-inducing agents are used for the management of cancer [12]. Eukaryotic cell cycle progression involves the sequential activation of cyclin-dependent kinases (CDKs) whose activation is dependent upon their association with cyclins [13]. Progression through the mammalian mitotic cycle is controlled by multiple holoenzymes comprising a catalytic CDK and a cyclin regulatory subunit [13]. These cyclin-CDK complexes are activated at specific intervals during the cell cycle but can also be induced and regulated by exogenous factors. Cell cycle progression is also regulated by the relative balance between the cellular concentrations of CDK inhibitors, including p27/KIP and p21/WAF1 [14]. The cyclin-CDK complexes are subjected to inhibition via binding with CDK inhibitors [14].

Cancer metastasis, the spread of cancer cells from the primary neoplasm to distant sites and their growth there, is the major cause of death in various cancer patients [15]. Metastasis of cancer cells involves multiple processes and various cytophysiological changes, including changes in adhesion capability between cells and extracellular matrix and abnormal intercellular interaction. Thus, degradation of the extracellular matrix and components of the basement membrane caused by concerted action of proteinases, such as matrix metalloproteinases (MMPs), cathepsins and plasminogen activator (PA), plays a critical role in tumor invasion and metastasis [16]. Among these enzymes, MMP-2, MMP-9 and urokinase plasminogen activator (u-PA) can directly degrade most components of the ECM and are deeply involved in cancer invasion and metastasis [17, 18]. Therefore, the inhibition of migration or invasion mediated by MMP-2, MMP-9 or u-PA could be a way to prevent or inhibit cancer metastasis [17].

Cervical cancer, a slow growing squamous cell carcinoma, is a common disease in women. Carcinoma of the uterine cervix is one of the highest causes of mortality in women cancer patients worldwide, and due to non-existent or inadequate screening, the disease is normally detected in the late stage. For 2000 years, medicinal herbs have been used in China to improve health and longevity. Studies utilizing a variety of chemical or biological interventions demonstrated promising results for induction of cell cycle arrest/apoptosis and inhibition of metastasis in malignant cells. Previous investigation demonstrated that ethanol (70%) AP extracts exhibit antiproliferative effects by induction of apoptosis in human squamous carcinoma KB cells [19]. Thus, we investigated the anticancer effects of AP extracts (25–200 *μ*g/mL) in terms of tumor regression using both *in vitro* cell culture and *in vivo* athymic nude mice models of KB cells. The levels of cell cycle/metastasis control and related molecules were assayed to determine the *A. pricei* anticancer mechanism.

## 2. Methods

### 2.1. Chemicals

Dulbecco's Modified Eagle's medium (GIBCO BRL, Grand Island, NY), antibody against cyclin B1, Cdc2, p21/WAF1, Wee1, p53, phospho-p53 (p-p53), MMP-9, u-PA, u-PA receptor (u-PAR), tissue inhibitor of MMP (TIMP-1) and PAI-1 (Santa Cruz Biotechnology, Inc., Heidelberg, Germany), antibody against *β*-actin (Sigma Chemical Co., St. Louis, MO), and antibody against cyclin A, Cdc25C (Cell Signaling Technology Inc., Danvers, MA) were obtained from their respective suppliers. All other chemicals were of the highest grade commercially available and supplied either by Merck (Darmstadt, Germany) or Sigma.

### 2.2. Preparation of AP Extracts

The roots of *A. pricei* were collected in March 2007 in Ping-tung County, an administrative region located in southern Taiwan, and were identified by Dr Yen-Hsueh Tseng (National Chung Hsing University, Taichung) [19]. The voucher specimen was deposited in the herbarium of the same university. The air-dried roots (2 kg) of *A. pricei* were extracted with 10 l of 70% ethanol at room temperature. The crude extracts (166 g) were concentrated in a vacuum and freeze dried to form a powder. The stock was then stored at −20°C for subsequent analysis of its anticancer properties. The yield of AP extract was 8.3%. For preparation of the stock solution, the powder samples were dissolved in 70% ethanol at 25°C.

### 2.3. Cell Culture and Assessment of Cell Growth and Viability

The human carcinoma cell line, KB (HeLa derivative), was obtained from the American type Culture Collection (Rockville, MD). The KB cell line was used by the National Cancer Institute, USA for some of the earliest *in vitro* anticancer drug-screening work. They were originally thought to be derived from an epidermoid carcinoma of the oral cavity [20]. In fact, they were derived from a glandular cancer of the cervix [21]. These cells were grown in DMEM supplemented with 10% heat-inactivated fetal bovine serum, 2 mM glutamine and 1% penicillin-streptomycin-neomycin in a humidified incubator (5% CO_2_ in air at 37°C). Cells were seeded in 6- or 12-well plates prior to the addition of AP extracts. The KB cells were incubated with AP extracts (0–200 *μ*g/mL) for 24, 48 and 72 h. Cultures were harvested and monitored for cell number by counting cell suspensions in a hemacytometer. Cell viability (3.0 × 10^5^ cells/12-well dish) and growth (1.0 × 10^5^ cells/6-well dish) were checked before and after treatment with AP extracts using trypan blue exclusion and were examined using phase contrast microscopy.

### 2.4. Flow Cytometric Analysis

Cellular DNA content was determined by flow cytometric analysis of propidium iodide (PI)-labeled cells [22]. KB cells were grown to exponential phase, seeded at a density of 1 × 10^6^ cells/60-mm dish, and treated with the indicated concentrations of AP extracts (0–200 *μ*g/mL) for 24 h. After treatment, the cells were collected by trypsinization, and fixed in ice-cold 70% ethanol at −20°C overnight. The cells were resuspended in phosphate buffered saline (PBS) containing 1% Triton X-100, 0.5 mg/mL of RNase, and 4 *μ*g/mL of PI at 37°C for 30 min. A FACSCalibur flow cytometer (Becton Dickinson, San Jose, CA) equipped with a single argon-ion laser (488 nm) was used for flow cytometric analysis. Forward and right-angle light scatter, which correlated with the size of the cell and the cytoplasmic complexity, respectively, were used to establish size gates and exclude cellular debris from the analysis. The DNA content of 10 000 cells per analysis was monitored using the FACSCalibur system. The cell cycle was determined and analyzed using ModFit software (Verity Software House, Topsham, ME).

### 2.5. Preparation of Total Cell Extract and Immunoblot Analysis

KB cells (3.0 × 10^6^ cells/100 mm dish) were detached, washed once in cold PBS, and then suspended in 100 *μ*L lysis buffer (10 mM Tris–HCl (pH 8), 0.32 M sucrose, 1% Triton X-100, 5 mM EDTA, 2 mM dithiothreitol and 1 mM phenylmethyl sulfonyl fluoride). The suspension was put on ice for 20 min and then centrifuged at 5000 rpm for 20 min at 4°C. Total protein content was determined using Bio-Rad protein assay reagent, with bovine serum albumin as the standard; protein extracts were reconstituted in sample buffer (0.062 M Tris-HCl, 2% sodium dodecylsulfate (SDS), 10% glycerol and 5% *β*-mercaptoethanol), and the mixture was boiled for 5 min. Equal amounts (50 *μ*g) of the denatured proteins were loaded into each lane, separated on 8–15% SDS polyacrylamide gels, followed by transfer of the proteins to polyvinylidene difluoride (PVDF) membranes overnight. Membranes were blocked with 0.1% Tween-20 in PBS containing 5% non-fat dried milk for 20 min at room temperature, and the membranes were reacted with primary antibodies for 2 h. They were then incubated with either a horseradish peroxidase-conjugated goat anti-rabbit or anti-mouse antibody for 2 h before being developed using SuperSignal ULTRA chemiluminescence substrate (Pierce, Rockford, IL). Band intensities were quantified by densitometry, with absorbance of the mixture at 540 nm determined using an enzyme-linked immunosorbent assay (ELISA) plate reader. Western blot analyses, with antibodies against cyclin A, cyclin B1, Cdc2, Cdc25C, p21/WAF1, Weel, p53, p-p53, MMP-9, u-PA, u-PAR, TIMP-1 and PAI-1 were performed as described previously [22].

### 2.6. Determination of MMP-9 by Zymography

The activities of MMP-9 in the medium were measured by gelatin zymography protease assays [16, 17]. Briefly, collected media of an appropriate volume (adjusted by vital cell number) were prepared with SDS sample buffer without boiling or reduction and were subjected to 0.1% gelatin–8% SDS-PAGE electrophoresis. After electrophoresis, gels were washed with 2.5% Triton X-100 and then incubated in a reaction buffer (40 mM Tris-HCl, pH 8.0; 10 mM CaCl_2_ and 0.01% NaN_3_) at 37°C for 12 h. Then, the gels were stained with Coomassie brilliant blue R-250.

### 2.7. Animals

Female athymic nude mice (BALB/*c-nu*), 5–7 weeks of age, were purchased from GlycoNex, Inc., Taiwan, and were maintained in caged housing in a specifically designed pathogen-free isolation facility with a 12/12 h light/dark cycle; the mice were provided rodent chow and water *ad libitum*. All experiments were conducted in accordance with the guidelines of the China Medical University Animal Ethics Research Board.

### 2.8. Tumor Cell Inoculation

KB cells (1 × 10^6^ cells) were mixed in a 200 *μ*L matrix gel and then injected subcutaneously on the right hind flank. The components of matrix gel include growth factors. These cells were grown in DMEM medium supplemented with 10% heat-inactivated fetal bovine serum, 2 mM glutamine, 1% penicillin-streptomycin-neomycin in a humidified incubator (5% CO_2_ in air at 37°C). Experiments were performed using cells from fewer than 20 passages. Tumor volume, as determined by caliper measurements of tumor length, width and depth, were calculated using the formula: length × width^2^ × 1/2 every 3 days [23]. The two groups received intraperitoneal injections of AP extracts (0.2 mL/mouse) dissolved in PBS buffer at a dose of 10 mg/kg every 2 days, while the control group received daily injections of vehicle only. Following 27 days of treatment, the mice were sacrificed. The tumors were removed before fixing in 4% paraformaldehyde, sectioning and staining with hematoxylin-eosin for light microscopy. Part of the tumor tissue was immediately frozen and the rest was fixed in 10% neutral-buffered formalin and embedded in paraffin. To monitor drug toxicity, the body weight of each animal was measured every 3 days. In addition, a pathologist examined the mouse organs, including liver, lungs and kidneys.

### 2.9. In Situ Apoptosis Detection

Apoptotic cell death in deparaffinized tissue sections was detected using terminal deoxynucleotidyl transferase-mediated dUTP nick end-labeling (TUNEL) with the Klenow DNA fragmentation detection kit (Calbiochem, San Diego, CA) [24]. Briefly, sections were permeabilized with 20 *μ*g/mL protease K in Tris-buffered saline, and endogenous peroxidase was inactivated by 3% H_2_O_2_ in methanol. Apoptosis was detected by labeling the 3′-OH ends of the fragmented DNA with biotin-dNTP using the Klenow at 37°C for 1.5 h. The slides were then incubated with streptavidin horseradish peroxidase conjugate, followed by incubation with 3,3′-diaminobenzidine and H_2_O_2_. Apoptotic cells were identified by the dark brown nuclei observed with light microscopy.

### 2.10. Statistical Analyses

*In vitro* results are presented as mean ± standard deviation (SD). For *in vivo* experiments, mean data values are presented with standard error (SE). All study data were analyzed using analysis of variance, followed by Dunnett's test for pair-wise comparison. Statistical significance was defined as *P* <  .05 for all tests.

## 3. Results

This study investigated the anticancer effects of ethanol (70%) extracts of *A. pricei* rhizomes both *in vitro* and *in vivo* using the KB epidermoid carcinoma cell line and nude mice xenograft models.

### 3.1. G2/M Arrest in AP Extracts-Treated KB Cells

**Figure 1:**
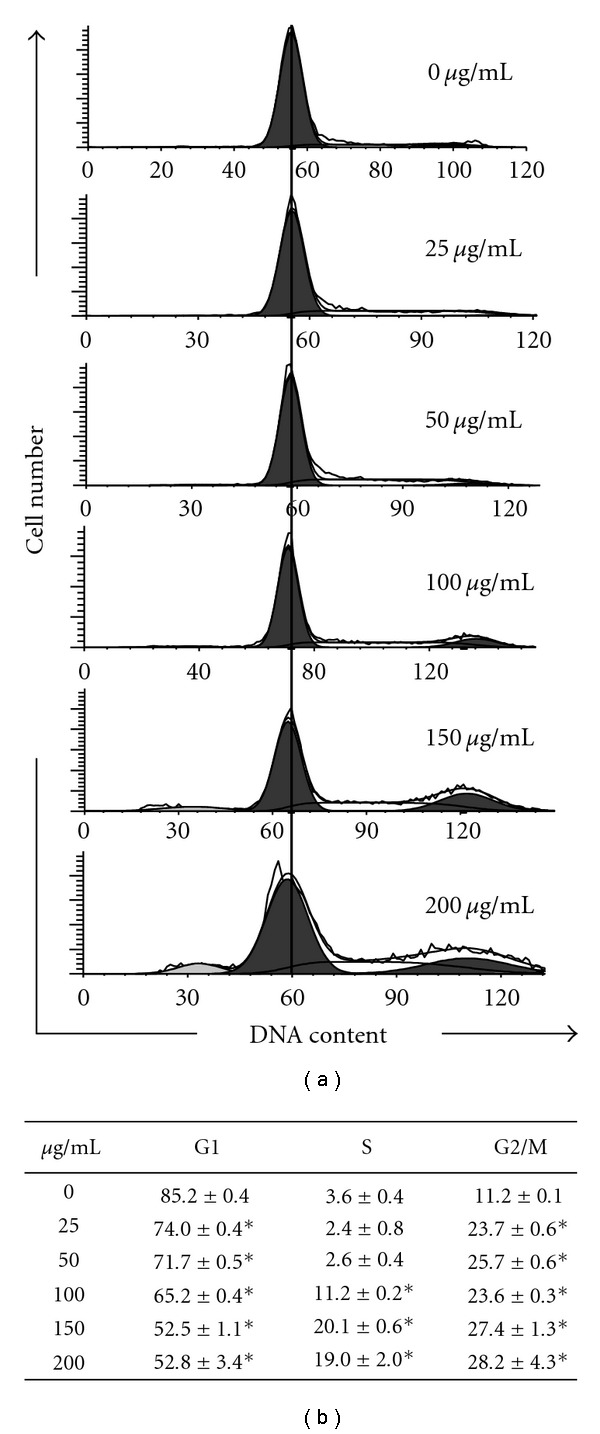
Effects of *A. pricei* (AP) extracts on KB cell cycle distribution. (a) Cells were treated with 0, 25, 50, 100, 150 or 200 *μ*g/mL of AP extracts for 24 h, stained with PI, and analyzed for cell cycle phase using flow cytometry. (b) Cellular distribution (percentage) in different phases of the cell cycle (G1, S and G2/M) after AP extract treatment. Results are presented as mean ± SD of three assays. *Significant difference in comparison to control group (*P* <  .05).

The profile of the DNA content of the AP extract-treated KB cells (0–200 *μ*g/mL for 24 h) was obtained using flow cytometric analysis to measure the fluorescence of PI-DNA binding. The stage at which AP extract-induced growth inhibition occurred in KB cell cycle progression was determined, along with cellular distributions in the different phases after treatment. Figures 1(a) and 1(b) show that AP extract exposure resulted in progressive and sustained accumulation of cells in the G2/M phase in a dose-dependent manner. Furthermore, the percentage of S and G2/M phase cells increased, while those in the G1 phase decreased after treatment with AP extracts (Figures 1(a) and 1(b)). Our findings suggest that *A. pricei* promotes cell growth inhibition by inducing G2/M phase arrest in cancer cells.

### 3.2. Western Blot Analysis of Cyclin A, Cyclin B1, Cdc2 and Cdc25C Levels in KB Cells after Exposure to AP Extracts

**Figure 2:**
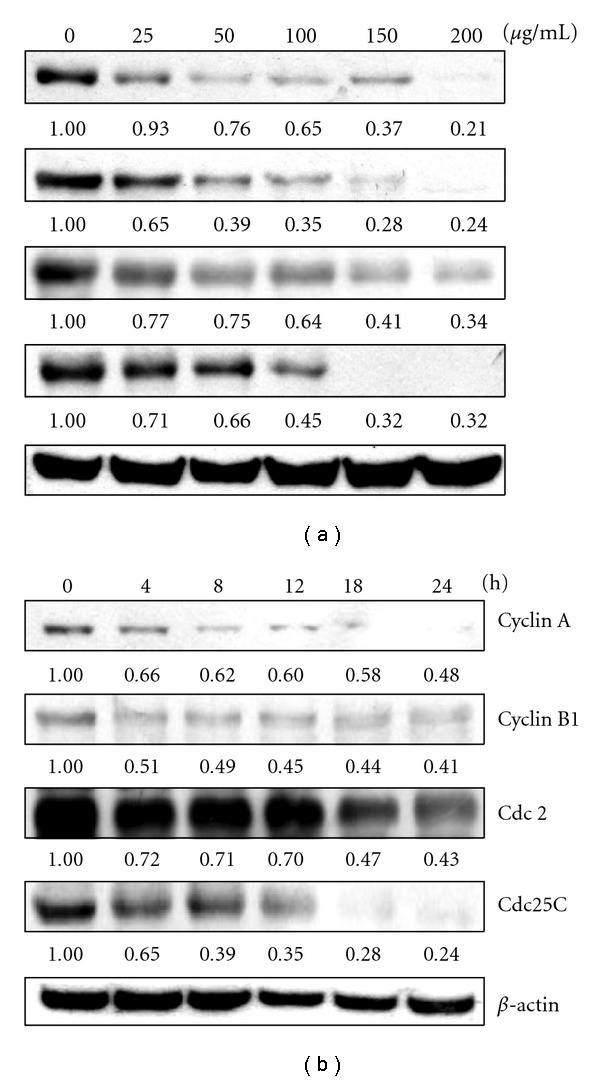
Western blot analysis of cyclin A, cyclin B1, Cdc2 and Cdc25C protein levels in KB cells after exposure to AP extracts. (a) Cells were treated with 0, 25, 50, 100, 150 or 200 *μ*g/mL of AP extracts for 24 h. (b) Cells were treated with 150 *μ*g/ml of AP extracts for 0, 4, 8, 12, 18 or 24 h. Protein (50 *μ*g) from each sample was resolved on 8–12% SDS–PAGE and western blotting was performed. *β*-actin was used as a control. Relative changes in protein bands were measured using densitometric analysis. Typical results from three independent experiments are shown.

In order to examine the molecular mechanism(s) and underlying changes in cell cycle patterns, we investigated the effects of various cyclins and CDKs involved in cell cycle control of KB cells. We approached this study by treating KB cells with AP extracts (0–200 *μ*g/mL) for 0–24 h. Dose- and time-dependent reductions in mitotic cyclins A and B1, mitotic-cyclin dependent kinase Cdc2, and mitotic phosphatase Cdc25C expression with treatment were observed (Figures 2(a) and 2(b)). This result implies that AP extracts inhibited cell cycle progression by reducing cyclin A, cyclin B1, Cdc2 and Cdc25C.

### 3.3. AP Extracts Increase the Expression of p21/WAF1, Wee1, p53 and p-p53

**Figure 3:**
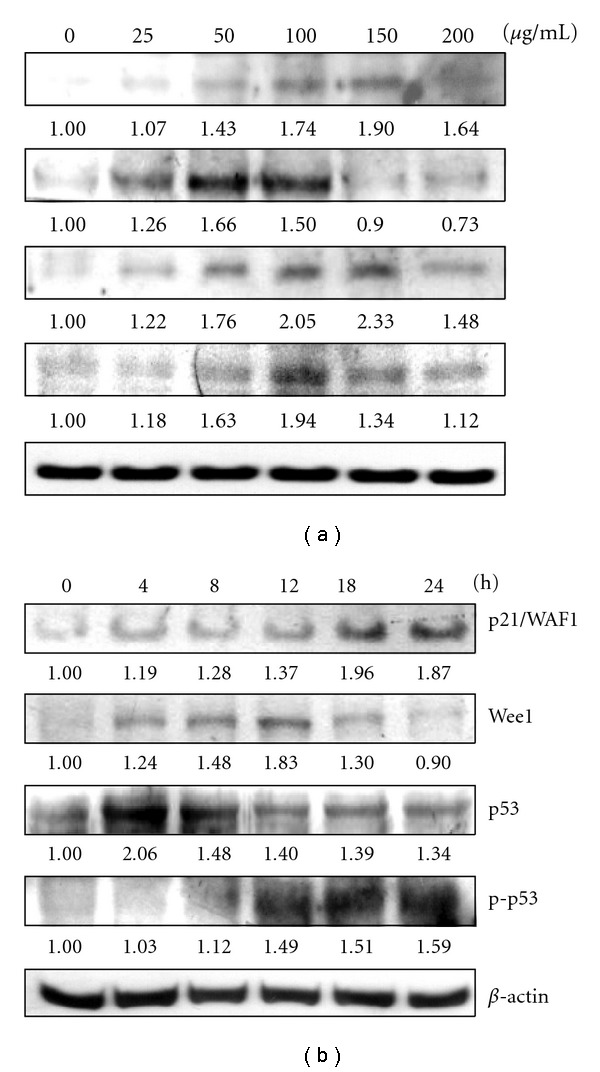
Western blot analyses of p21/WAF1, Weel, p53 and p-p53 protein levels in KB cells after exposure to AP extracts. (a) Cells were treated with 0, 25, 50, 100, 150 or 200 *μ*g/mL of AP extracts for 24 h. (b) Cells were treated with 150 *μ*g/mL of AP extracts for 0, 4, 8, 12, 18 or 24 h. Protein (50 *μ*g) from each sample was resolved on 8–15% SDS-PAGE and western blotting was performed. *β*-actin was used as a control. Relative changes in protein bands were measured using densitometric analysis. Typical results from three independent experiments are shown.

As shown in our study, AP extract treatment of KB cells resulted in cell cycle arrest. Thus, we also examined the effect of exposure on cell cycle-regulatory molecules, including p21/WAF1 (CDK inhibitors), Weel (CDK relative factors), p53 and p-p53. Figures 3(a) and 3(b) show that treatment of KB cells with AP extracts (0–200 *μ*g/mL for 0–24 h) induced marked dose and time-dependent up-regulation of p21/WAF1, Weel, p-p53 and p53 protein expression.

### 3.4. Effects of AP Extracts on Levels of MMP-9, u-PA, u-PAR, TIMP-1 and PAI-1, and on Activity of MMP-9

**Figure 4:**
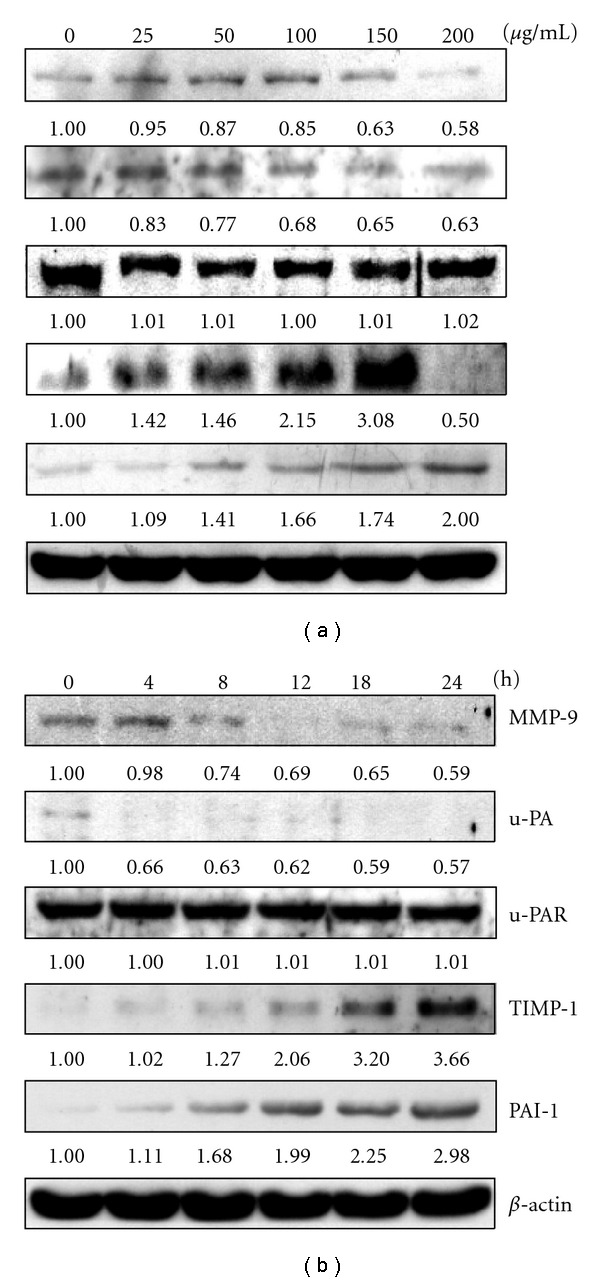
Western blot analysis of MMP-9, u-PA, u-PAR, TIMP-1 and PAI-1 protein levels in KB cells after exposure to AP extracts. (a) Cells were treated with 0, 25, 50, 100, 150 or 200 *μ*g/mL of AP extracts for 24 h. (b) Cells were treated with 150 *μ*g/mL of AP extracts for 0, 4, 8, 12, 18 or 24 h. Protein (50 *μ*g) from each sample was resolved on 8–15% SDS-PAGE and western blotting was performed. *β*-actin was used as a control. Relative changes in protein bands were measured using densitometric analysis. Typical results from three independent experiments are shown.

**Figure 5:**
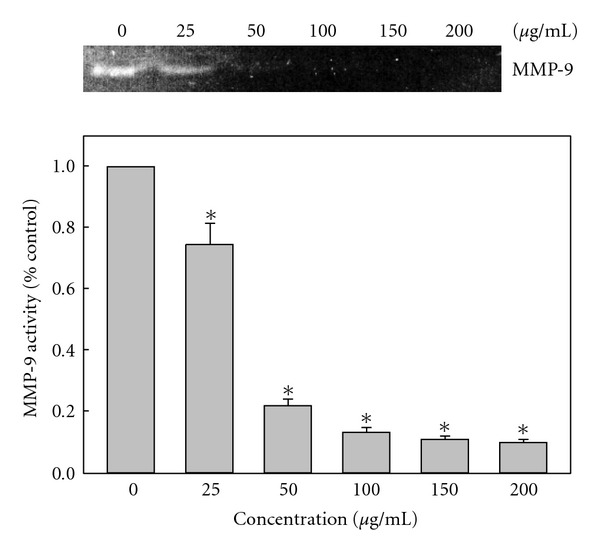
Effects of AP extracts on the activities of MMP-9. KB cells were treated with 0, 25, 50, 100, 150 or 200 *μ*g/mL of AP extracts for 24 h and then subjected to gelatin zymography to analyze the activities of MMP-9. Relative changes in protein bands were measured using densitometric analysis. Typical results from three independent experiments are shown. Results are presented as mean ± SD of three assays. *Significant difference in comparison to control group (*P* <  .05).

We also investigated whether AP extracts inhibited metastasis-related proteins in KB cells. We used western blotting to analyze the effects of AP extracts on MMP-9, u-PA, u-PAR, TIMPs and PAI-1 expression. As shown in Figure 4, treatment of KB cells with AP extracts (0–200 *μ*g/mL for 0–24 h) induced marked dose- and time-dependent reductions in MMP-9 and u-PA protein expression. Dose- and time-dependent up-regulation of the specific endogenous inhibitors, TIMP-1 and PAI-1 expression with AP extract treatment were observed (Figures 4(a) and 4(b)). However, the experimental treatment did not appear to change the amount of detectable u-PAR (u-PA receptor) protein (Figures 4(a) and 4(b)). Moreover, gelatin zymography assays showed that AP extracts (0–200 *μ*g/mL for 24 h) reduced MMP-9 activity in a dose-dependent manner in KB cells (Figure 5).

### 3.5. *In Vivo* Inhibition of KB Xenograft Proliferation by AP Extracts

**Figure 6:**
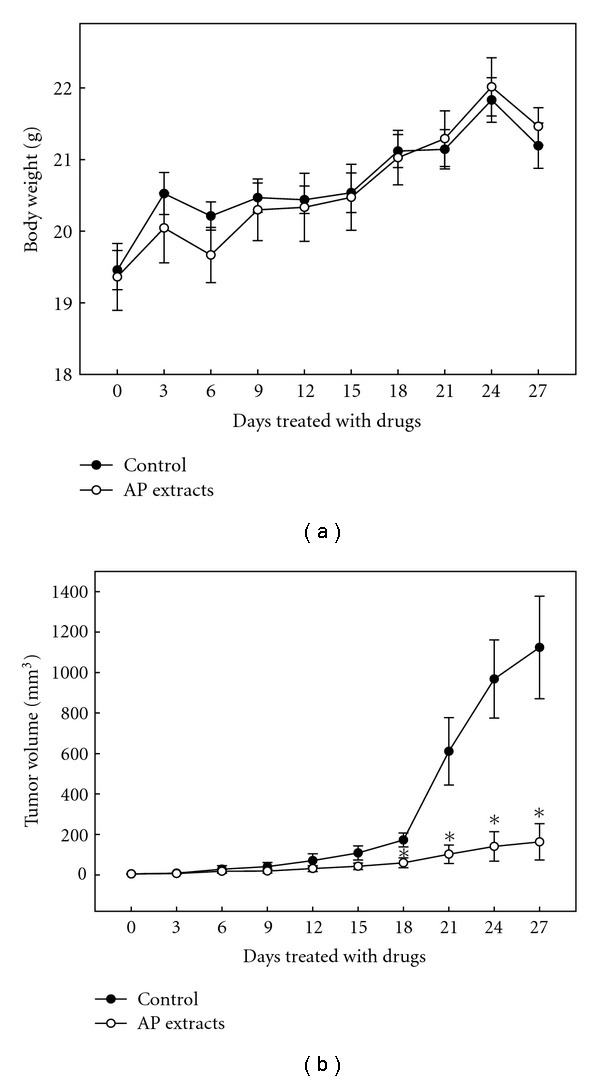
Time-course effect of AP extracts on growth of KB xenografted nude mice was evaluated by measurements of body weight (a) and tumor volume (b) every 3 days. KB cells were implanted subcutaneously into the flanks of nude mice on Day 0, the animals were then treated with 10 mg/kg of AP extracts or were untreated (control). Results are presented as mean ± SE (*n* = 6). *Significant difference in comparison to control group (*P* <  .05).

**Figure 7:**
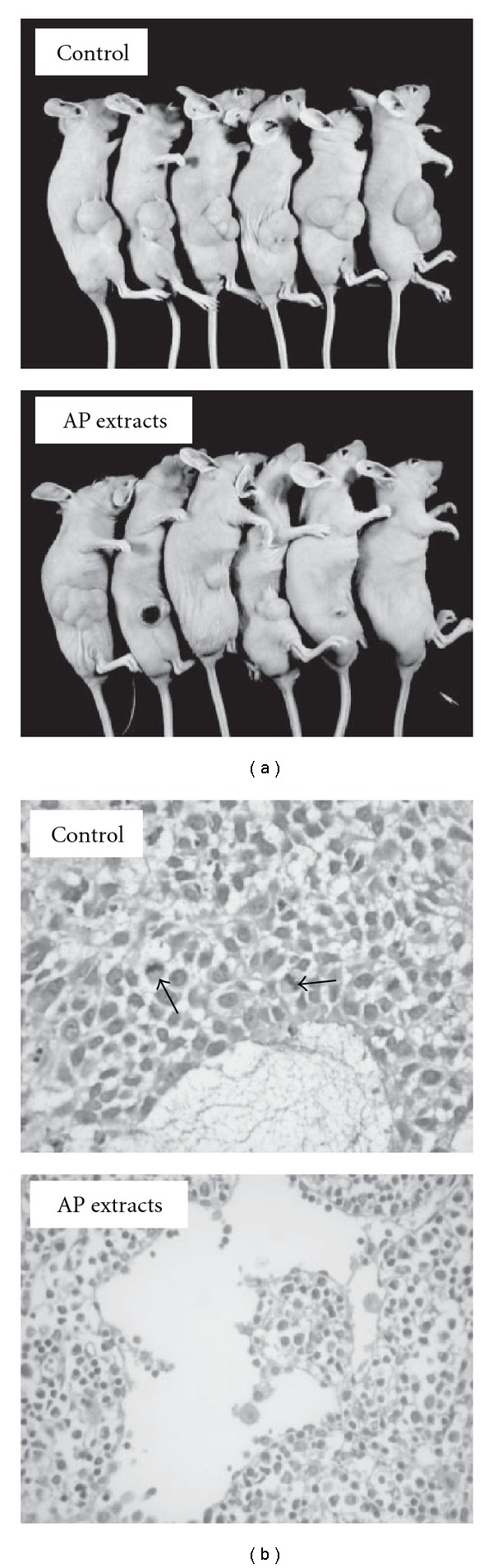
*In vivo* inhibition of KB xenograft proliferation by AP extracts. (a) Nude mice were untreated (control) or treated with 10 mg/kg of AP extracts. On the 27th day after tumor implantation, the animals were sacrificed and the tumors were removed. (b) The control KB xenograft tumor and the KB xenograft tumors after AP extract treatment were sectioned and stained with hematoxylin and eosin, examined with light microscopy. Arrows indicate mitosis-positive cells. Typical results from three independent experiments are shown.

Nude mice were used to evaluate the *in vivo* effects of AP extracts on tumor growth. KB cells were xenografted into nude mice as described in “Methods” section. All of the animals appeared healthy, with no loss of body weight noted during AP extract treatment (Figure 6(a)). In addition, no signs of toxicity were observed (body weight and microscopic examination of individual organs; data not shown) in any of the nude mice. The time course for KB xenograft growth with AP extracts (10 mg/kg/every 2 days) or without treatment (control) is shown in Figures 6(b) and 7(a). Tumor volume in the AP extracts-treated mice was inhibited as compared with the control group (Figure 7(a)). Evaluation of tumor volume showed significant dose- and time-dependent growth inhibition associated with AP extract treatment (Figure 6(b)). At the end of 27 days, the KB xenograft tumor was excised from each sacrificed animal. Additionally, microscopic examination of the tumor sections was done to distinguish differences in nucleic and cytoplasmic morphology after the 27 days of AP extract treatment. Furthermore, abundant mitosis was observed in the proliferating cells in the control group, while the mitosis-positive cells decreased in sections from the treated animals (Figure 7(b)). These results demonstrated AP extract-related antitumor activity in nude mice bearing KB epidermoid carcinoma xenografts.

### 3.6. Induction of Apoptotic DNA Fragmentation by AP Extracts in Xenograft Tumors

**Figure 8:**
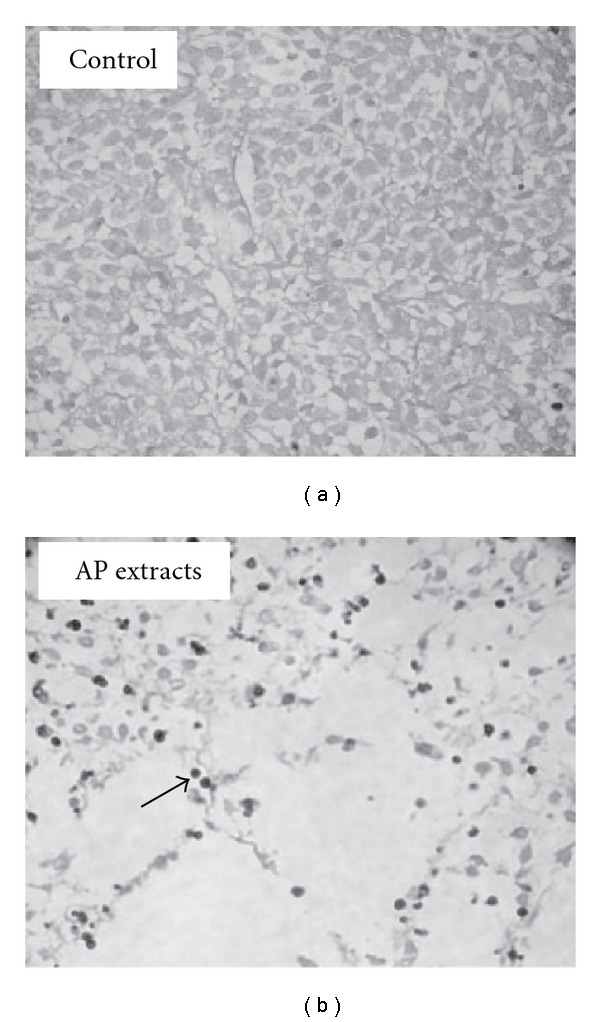
Immunohistochemical staining of apoptotic DNA fragmentation in KB xenograft tumors. *In situ* apoptosis detection using TUNEL staining in tumor sections from control animals (a) and experimental analogs treated with AP extracts (10 mg/kg) (b). Arrow indicates apoptotic-positive cells. Typical results from three independent experiments are shown.

The effect of AP extracts on tumor growth (apoptosis) in the KB xenograft mice was also examined using the TUNEL assay on the tumor sections. Figures 8(a) and 8(b) show that there were more TUNEL-positive cells in tumors from AP extract-treated animals, relative to that of the untreated controls. These results demonstrated that AP extract treatment was associated with decreased proliferation and increased apoptosis in the study animals. Analysis of our data suggests that AP extracts promoted antitumor activity in nude mice bearing KB epidermoid carcinoma xenografts.

## 4. Discussion

Herbal medicine is one of the most ancient forms of health care known to humankind and it has been used in many cultures throughout history. Several studies have demonstrated anticancer potential for extracts from a number of herbal medicines or mixtures *in vitro* or *in vivo*. Typically, herbal medicines emphasize the use of whole extracts from a plant mix or from complex formulations [25]. Anticancer agents may alter regulation of the cell cycle machinery, resulting in cellular arrest at different phases of the cell cycle and, thereby, reducing the growth and proliferation of, and even inducing apoptosis in, cancerous cells. The present research documents a parallel study showing the effect of AP extract treatment *in vivo* in a human tumor xenograft in nude mice and *in vitro* in a cell culture model involving human squamous carcinoma KB cells. From those results, we determined that AP extract arrests KB cells at the G2/M phase by (i) inhibiting protein levels of cyclin A and cyclin B1, (ii) decreasing Cdc2 and Cdc25C levels, (iii) increasing p21/WAF1, Wee1, p53 and p-p53 levels, (iv) decreasing MMP-9 and u-PA, and increasing their endogenous inhibitors, TIMP-1 and PAI-1. Moreover, our previous investigation has demonstrated that AP extracts exhibit an antiproliferative effect by induction of apoptosis in KB cells [19]. Studies in the past decade have shown that concurrent chemotherapy programs have the potential to improve the overall survival of patients with squamous cell carcinoma. Interestingly, *A. pricei* has been found to have lesser cytotoxicity towards the normal human fibroblast (HGF) cells [19]. These results suggest that *A. pricei* may be useful for the prevention and/or treatment of patients with cancer. Furthermore, *in vivo* toxicity was also examined superficially from body weight changes and histological studies of vital organs (data not shown). There appeared to be no signs of significant toxicity at *A. pricei* exposures of 10 mg/kg. This likely indicates that there are no side effects at these doses. Investigation of the nontoxic characteristics of *A. pricei* in rats is necessary, which may increase its potential for application in food and drug products. Future studies should test whether there is an optimal/effective dose for *A. pricei* exposure. Summary of our data suggests that treatment with AP extracts could be effective in suppressing the proliferation of KB cells as shown by growth inhibition, cell cycle arrest, and apoptosis induction *in vivo* or *in vitro*.

Disturbance of the cancer cell cycle is one of the therapeutic targets for development of new anticancer drugs [26]. Several studies have reported that various cytotoxic drugs can induce G2/M phase arrest [27]. The results of cell cycle analysis in the present study, as evaluated by flow cytometry, show that the AP extract treatment had a profound effect on cell cycle control, with squamous carcinoma cells accumulating in the G2/M phase. This cell cycle blockade was associated with reductions in cyclin A, cyclin B1, Cdc2 and Cdc25C, and increased CDK inhibitor p21/WAF1, Weel, p53 and p-p53. Eukaryotic cell cycle progression involves the sequential activation of CDK, whose activation is dependent upon association with cyclins. Among CDKs that regulate cell cycle progression, CDK2 and Cdc2 kinases are primarily activated in association with cyclin A and cyclin B1 during progression of the G2/M phase [28, 29]. The phosphorylation of Tyr15 of Cdc2 suppresses activity of Cdc2/cyclin A and B1 kinase complex. Dephosphorylation of Tyr15 of Cdc2 is catalyzed by Cdc25C phosphatase, and this reaction is believed to be the rate-limiting step for entry into mitosis [30]. P53 could act as a sensor for DNA damage that could arrest the cell cycle for DNA repair or upregulate pro-apoptotic factors resulting in increased susceptibility to apoptosis. Following DNA damage, the CDK inhibitor p21/WAF1 is expressed in p53-dependent or p53-independent manner [31]. P21/WAF1 may help to maintain G2/M cell cycle arrest by inactivating cyclin B1/Cdc2 complex, disrupting the interaction between proliferating cell nuclear antigen and Cdc25C [31]. The results imply that the expression of cyclin A, cyclin B, Cdc2 and Cdc25C are down-regulated, and CDK inhibitors p21/WAF1, Weel, p53 and p-p53 are increased in AP extract-treated KB cells, which corroborates G2/M block. Analysis of our data suggests that the observed inhibition of proliferation of KB cells associated with AP extract treatment could be the result of cell cycle arrest during the G2/M phase.

Metastasis is the spread of cancer cells from the primary tumor to the new metastatic sites via blood or lymph vessels [32]. MMPs and u-PA, which are secreted by invasive cancer cells, play important roles in cancer cell invasion and metastasis because tumor cells must cross type IV collagen-rich basement membrane of vessel walls to spread to other sites during a period of cancer metastasis [32]. *In vivo* evidence from the chicken chorionallantoic membrane assay showed that MMP-9 and u-PA are interdependent in tumor invasion, which also showed that invasion was dependent on both MMP-9 and u-PA, because in the absence of MMP-9 and u-PA, tumor cells showed only low levels of invasion [33]. Therefore, inhibition of invasion mediated by MMP-9 and u-PA may be a key feature for the prevention of cancer metastasis. Physiological activities of MMPs and u-PA were greatly related to that of TIMP and PAI, respectively, their specific endogenous inhibitors. Furthermore, TIMPs play a key role in determining the proteolytic activity of tumor tissues by regulating the activity of MMPs. Accordingly, several studies demonstrated correlation between high invasiveness and decreased MMP-2 expression [34, 35]. Conversely, the anti-invasive action of several anticarcinogenic substances is associated with elevated MMP-1 levels [36]. PAIs (serine protease inhibitors) regulate uPA and tissue plasminogen activator (tPA) to control plasmin generation. PAI is implicated as a mediator of invasion and metastasis in several tumor types [37]. There is increasing evidence for PAI in cancer metastasis independent of its protease inhibitory activity, such as tumor cell motility, cell and anti-cell adhesion, pro- and anti-angiogenesis, and prevention of apoptosis [37]. In cervical cancer, increased levels of uPA and PAI-1 proteins were detected in cervical tumor extracts but not in normal tissue extracts [38] and are linked to poor prognosis [39]. In this study, we revealed that AP extracts decreased protein levels of tumor metastasis-related proteins such as MMP-9 and u-PA in KB cells. Meanwhile, their endogenous inhibitors, which are TIMP-1 and PAI-1, were increased in KB cells. It has been shown that KB cells exhibited a reduced motility and also reflected fewer invasions without altering the MMP status [40]. Therefore, the inhibition of KB cell migration and invasion by AP extracts was not examined in this study.

Our previous investigation demonstrated that AP extract exhibits an antiproliferative effect by induction of apoptosis that is associated with loss of cell viability, morphologic change, internucleosomal DNA fragmentation, sub-G1 phase accumulation, loss of mitochondrial membrane potential (ΔΨm), cytochrome *c* translocation, caspase-3 and -9, activation, polyadenosine diphosphate-ribose polymerase (PARP) degradation, dysregulation of Bcl-2 and Bax, and generation of reactive oxygen species (ROS) in KB cells [19]. Apoptosis is an important homeostatic mechanism that balances cell division and cell death and maintains the appropriate number of cells in the body. Disturbances of apoptosis in cancer cells have been studied in detail, and induction of apoptosis in these cells is one of the strategies for anticancer drug development [41]. Furthermore, tumor inhibition by AP extract was also observed in the nude mice xenograft model in this study. Both incidence and mean tumor volume were significantly reduced by AP extract treatment. Immunohistochemical staining revealed increased apoptosis (TUNEL assay) in tumors from the AP extract-treated animals. Analysis of our data suggests that AP extracts could induce apoptotic cell death in human squamous carcinoma KB cells both *in vitro* and *in vivo*.

Natural products, including plants, provide rich resources for anticancer drug discovery [42–44]. As the different components in a given herb may have synergistic activities or buffer toxic effects, mixtures or extracts of these herbs may offer greater therapeutic or preventive activity when used in combination [45]. In our study, a number of compounds, including desmethoxyyangonin, cardamonin, and flavokawain B were isolated from ethanol extracts of the *A. pricei* root [10]. These results imply that desmethoxyyangonin, cardamonin and flavokawain B could act as potential chemopreventive agents with respect to inhibition of the proliferation (growth) of human squamous carcinoma KB cells, through the induction of cell cycle arrest/apoptosis *in vitro* or *in vivo*. Further studies to identify the active constituents in *A. pricei* rhizome extracts leading to antitumor effects would be most important for future work.

**Figure 9:**
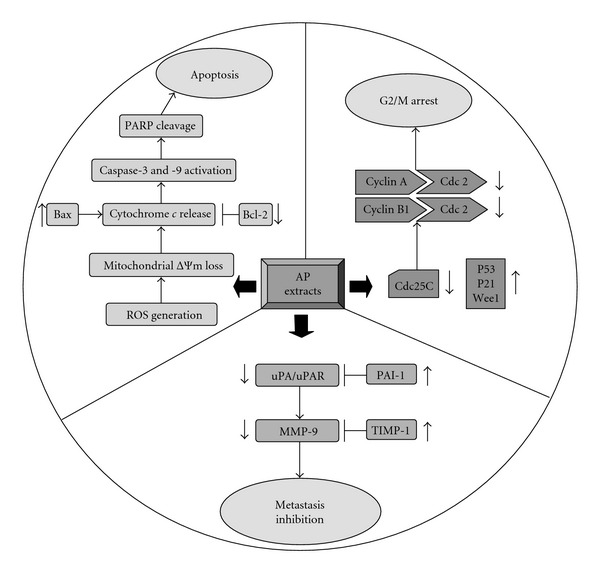
Proposed diagrams of AP extracts-induced apoptosis, G2/M arrest and metastasis inhibition in KB cells.

Based on the outcome of our studies, the mechanisms by which AP extracts-induced apoptosis, G2/M arrest and metastasis inhibition in KB cells are summarized in Figure 9. Our data suggest that extracts of *A. pricei* merit investigation as antitumor agents for treatment of human squamous carcinoma KB cells. These data impart important knowledge in the development of food and drug products for human use. Further *in vivo* studies using other animal models and humans are necessary to elaborate and exploit this nascent promise.

## Funding

NSC95-2320-B-039-024-MY2, CMU 96-207 and CMU 97-130 from the National Science Council and China Medical University of Taiwan.
